# COVID-19-associated coagulopathy: thromboembolism prophylaxis and poor prognosis in ICU

**DOI:** 10.1186/s40164-021-00202-9

**Published:** 2021-02-01

**Authors:** Runhui Zheng, Jing Zhou, Bin Song, Xia Zheng, Ming Zhong, Li Jiang, Chun Pan, Wei Zhang, Jiaan Xia, Nanshan Chen, Wenjuan Wu, Dingyu Zhang, Yin Xi, Zhimin Lin, Ying Pan, Xiaoqing Liu, Shiyue Li, Yuanda Xu, Yimin Li, Huo Tan, Nanshan Zhong, Xiaodan Luo, Ling Sang

**Affiliations:** 1 State Key Lab of Respiratory Diseases, Guangzhou Institute of Respiratory Health, Department of Pulmonary and Critical Care Medicine, The First Affiliated Hospital of Guangzhou Medical University, 510120 Guangzhou, China; 2 Hematology Department, The First Affiliated Hospital of Guangzhou Medical University, 510120 Guangzhou, China; 3 Department of Tuberculosis and Respiratory Disease, Wuhan Jinyintan Hospital, 430023 Wuhan, China; 4 Department of Critical Care Medicine, The First Affiliated Hospital of Zhejiang University, Hangzhou, Zhejiang China; 5 Department of Critical Care Medicine, Zhongshan Hospital Fudan University, Shanghai, China; 6 Department of Critical Care Medicine, Xuanwu Hospital, Capital Medical University, 100053 Beijing, China; 7 Department of Critical Care Medicine, Zhongda Hospital, Southeast University, 210009 Nanjing, China; 8 Emergency Department, The 900th Hospital of Joint Service Corps of Chinese PLA, 350025 FuZhou, China; 9 Department of Respiratory and Critical Care Medicine, Wuhan Jinyintan Hospital, Wuhan, China; 10 Department of Critical Care Medicine, Wuhan Jinyintan Hospital, Wuhan, China; 11 Research Center for Translational Medicine, Wuhan Jinyintan Hospital, Wuhan, China; 12 Joint Laboratory of Infectious Diseases and Health, Wuhan Institute of Virology and Wuhan Jinyintan Hospital, Chinese Academy of Sciences, 430023 Wuhan, Hubei China

**Keywords:** Coagulation parameters, COVID-19, D-dimer, Sepsis‐induced coagulopathy, Disseminated intravascular coagulation

## Abstract

**Background:**

Coronavirus disease 2019 (COVID-19) is associated with coagulation abnormalities which are indicators of higher mortality especially in severe cases.

**Methods:**

We studied patients with proven COVID-19 disease in the intensive care unit of Jinyintan Hospital, Wuhan, China from 30 to 2019 to 31 March 2020.

**Results:**

Of 180 patients, 89 (49.44 %) had died, 85 (47.22 %) had been discharged alive, and 6 (3.33 %) were still hospitalised by the end of data collection. A D-dimer concentration of > 0.5 mg/L on admission was significantly associated with 30 day mortality, and a D-dimer concentration of > 5 mg/L was found in a much higher proportion of non-survivors than survivors. Sepsis-induced coagulopathy (SIC) and disseminated intravascular coagulation (DIC) scoring systems were dichotomised as < 4 or ≥ 4 and < 5 or ≥ 5, respectively, and the mortality rate was significantly different between the two stratifications in both scoring systems. Enoxaparin was administered to 68 (37.78 %) patients for thromboembolic prophylaxis, and stratification by the D-dimer concentration and DIC score confirmed lower mortality in patients who received enoxaparin when the D-dimer concentration was > 2 than < 2 mg/L or DIC score was ≥ 5 than < 5. A low platelet count and low serum calcium concentration were also related to mortality.

**Conclusions:**

A D-dimer concentration of > 0.5 mg/L on admission is a risk factor for severe disease. A SIC score of > 4 and DIC score of > 5 may be used to predict mortality. Thromboembolic prophylaxis can reduce mortality only in patients with a D-dimer concentration of > 2 mg/L or DIC score of ≥ 5.

## Introduction

Coronavirus disease 2019 (COVID-19) is associated with coagulation abnormalities characterised by elevations in procoagulants, which are indicators of higher mortality. Critically ill patients with sepsis-induced coagulopathy (SIC) or disseminated intravascular coagulopathy (DIC) in the intensive care unit (ICU) account for the majority of deaths. Reports from Wuhan, China revealed increased D-dimer concentrations in 26–36 % of patients requiring ICU admission, and 71.4 % of non-survivors developed overt DIC [1–3]. Reports have suggested that the incidence of venous thromboembolism (VTE) is higher in ICU patients with severe COVID-19 than in patients in the wards and historically reported incidence rates of VTE in the ICU [4]. Anticoagulant treatment and outcomes are closely related to the SIC score and D-dimer concentration; therefore, the SIC criteria established by the International Society on Thrombosis and Haemostasis (ISTH) is often used to guide anticoagulant therapy [5, 6]. In an analysis of patients with severe COVID-19 from Tang, stratification by the SIC score revealed lower mortality in patients treated with prophylactic doses of heparin [2]. Many centres support increased prophylactic doses of anticoagulants for ICU patients because of the increased incidence of thrombotic complications despite the use of systematic thrombosis prophylaxis [7]. Therefore, coagulopathy management including monitoring of coagulation changes, thromboembolic prophylaxis, and anticoagulant treatment is becoming increasingly more important, and coagulopathy guidelines are needed to optimise specific therapy and reduce mortality.

In this study, we focused on patients with severe COVID-19 who were admitted to the ICU. Abnormal coagulation changes were studied, and the roles of both the SIC and DIC scoring systems in predicting mortality were evaluated. The incidence of VTE and the association between VTE prophylaxis and survival were investigated.

## Patients and methods

### Patients

Patients with proven COVID-19 disease who were admitted or transferred to the ICU of Jinyintan Hospital, Wuhan, China from 30 to 2019 to 31 March 2020 were retrospectively studied. COVID-19 was confirmed by reverse-transcription polymerase chain reaction. The indications of ICU admission were acute respiratory distress syndrome (ARDS), sepsis, severe arrhythmia or heart failure, and renal failure that requires kidney replacement therapy. Patients were followed until death, ICU discharge, or the end of data collection on 1 April 2020, whichever came first. This study was approved by the Medical Ethics Committee of Wuhan Infectious Disease Hospital (Approval No. KY-2020-56.01).

### Data collection

The patients’ medical history, including their history of cancer, diabetes, and VTE, was collected on admission. Blood test data from the day of admission to the ICU, confirmed VTE, and ICU discharge or death were also analysed. Clinical features including the body temperature, duration of low blood pressure (systolic blood pressure of < 90 mmHg and diastolic blood pressure of < 70 mmHg or any blood pressure level when vasoactive drugs were used), blood transfusion volume, and serum calcium concentration were collected. The lymphocyte count, platelet count, prothrombin time (PT), activated partial thromboplastin time (aPTT), fibrinogen concentration, D-dimer concentration, fibrin degradation product (FDP) concentration, and antithrombin concentration were routinely analysed. A lymphocyte count of < 1.0 × 10^9^/L and platelet count of < 150 × 10^9^/L were defined as lymphocytopenia and thrombocytopaenia, respectively. The ISTH Overt DIC and SIC scoring systems and the Chinese DIC scoring system (CDSS, version 2017) were applied to analyse the association between coagulation abnormalities and mortality [8] (Table 1).

**Table 1.** Scoring systems used to analyse associations of coagulation abnormalities and mortality

A. ISTH SIC scoring system		
Item	Score	Range
Platelet count (×10^9^/L)	1	100-150
	2	<100
PT-INR	1	1.2-1.4
	2	>1.4
SOFA score	1	1
	2	≥2

B. Chinese DIC scoring system	
Item	Score
Primary disease leading to DIC	2
Clinical manifestations	
Severe bleeding	1
Shock	1
Extensive cutaneous and mucosal embolism, or focal ischemic necrosis, or unexplained organ failure	1
Platelet count (×10^9^/L)	
Non-hematology malignancy	
≥100	0
80–< 100	1
<80	2
Decrease > 50% within 24h	1
Hematology malignancy	
< 50	1
Decrease > 50% within 24h	1
D-dimer (mg/L)	
< 5	0
5–< 9	2
≥9	3
PT and aPTT (sec)	
Prolongation of PT< 3 or aPTT< 10	0
Prolongation of PT≥3 or aPTT≥10	1
Prolongation of PT≥6	2
Fbg (g/L)	
≥1.0	0
< 1.0	1

*ISTH* International Society on Thrombosis and Haemostasis, *SIC* sepsis-induced coagulopathy, *PT-INR* prothrombin time–international normalised ratio, *SOFA* Sequential Organ Failure Assessment (SOFA score is the sum of four items: respiratory SOFA, cardiovascular SOFA, hepatic SOFA, and renal SOFA). *DIC* disseminated intravascular coagulation, *PT* prothrombin time, *aPTT* activated partial thromboplastin time, *Fbg* fibrinogen

### Thrombosis prophylaxis and anticoagulant treatment

For patients who received thrombosis prophylaxis, enoxaparin was administered at a dosage of 100 IU AXa/kg once daily for ≥ 5 days. For patients with suspected VTE or ultrasonography confirmed VTE, enoxaparin was administered at a dosage of 100 IU AXa/kg twice daily during hospitalisation. However, diagnostic tests were not performed in every patient with clinically suspected thrombotic complications because of limited medical resources.

### Statistical analysis

The proportions of ICU patients with thrombocytopaenia, overt DIC, a SIC score of > 4, an increased D-dimer concentration, and VTE were assessed. Parameters of coagulation and clinical features were compared between survivors and non-survivors and between patients with and without VTE using the chi-square test. Coagulation parameters including the PT, aPTT, fibrinogen concentration, D-dimer concentration, FDP concentration, antithrombin concentration, and platelet count on the day of admission to the ICU and on the day of ICU discharge or death were compared using the Wilcoxon signed rank test. Logistic regression and linear regression were used to analyse the associations among the D-dimer concentration, SIC or DIC score, and mortality. Kaplan–Meier survival curves were also plotted.

## Results

### Patients’ characteristics

In total, 200 patients with proven COVID-19 disease were categorised as ICU patients. Of these patients, we excluded 2 who died within 24 hours after admission, 7 who lacked information about coagulation parameters, and 11 who were undergoing extracorporeal membrane oxygenation therapy because such therapy promotes thrombosis and a hypercoagulable state. The remaining 180 patients (113 men, 67 women) were enrolled in the study. By the end of data collection, 89 (49.44 %) patients had died, 85 (47.22 %) had been discharged alive, and 6 (3.33 %) were still hospitalised. The patients’ characteristics and medical history are shown in Table 2. Their median age was 64 years, and patients aged ≥ 60 years had significantly increased mortality (*p* = 0.000). A history of cancer was found in only eight patients, and all of these patients died within 25 days after admission, indicating that cancer history could be a risk factor for mortality as reported [9, 10].

**Table 2.** Patients’ characteristics

Characteristics	Survivors (n=91)		Non-survivors (n=89)		*P* value
	No.	%	No.	%	
Age					0.000*
<60	46	25.56	19	10.56	
≥60	45	25.00	70	38.89	
Sex					0.335
Male	54	30.00	59	32.78	
Female	37	20.56	30	16.67	
Cancer history					0.003*
Yes	0		8	4.44	
No	91	50.56	81	45.00	
Diabetes history					0.156
Yes	13	7.22	20	11.11	
No	78	43.33	69	38.33	
VTE history					0.321
Yes	1	0.56	0		
No	90	50.00	89	49.44	

*VTE* venous thromboembolism

*Statistically significant

### Coagulation parameters: correlation with survival

**Fig. 1:**
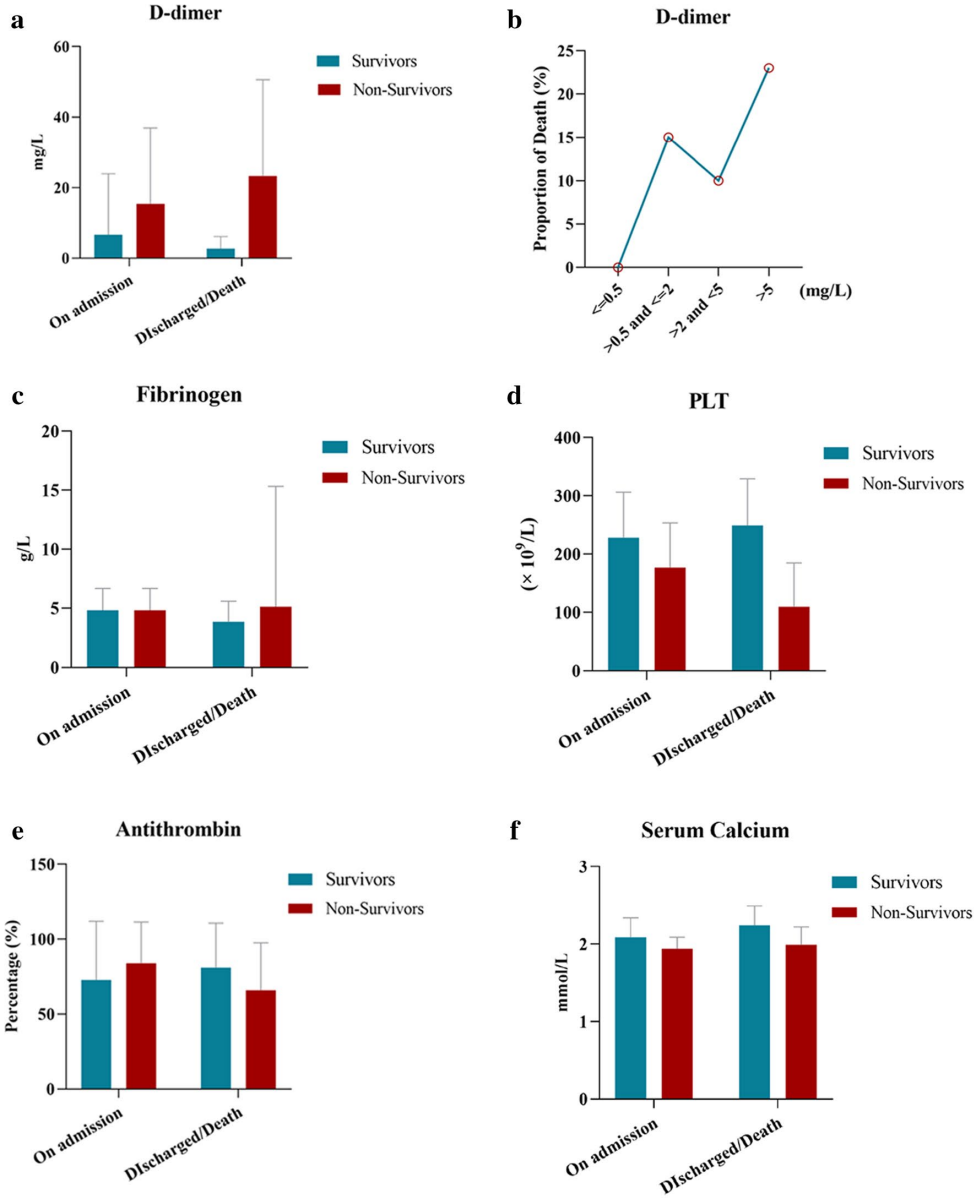
Comparison of coagulation parameters on the day of ICU admission versus the day of ICU discharge or death. **a** The D-dimer concentration was significantly increased in non-survivors on the day of death (*p* = 0.004). **b** The D-dimer concentration was stratified into four levels. The mortality rate was increased when the D-dimer concentration was > 0.5 mg/L and peaked when the D-dimer concentration was > 5 mg/L. **c** The fibrinogen concentration was increased in non-survivors and decreased in survivors on the day of ICU discharge or end of data collection (*p* = 0.001, 0.000). **d** The PLT was decreased in non-survivors on the day of death but increased as patients recovered (*p* = 0.000, 0.024). **e** The antithrombin concentration was significantly decreased in non-survivors on the day of death (*p* = 0.000). **f** The serum calcium concentration was decreased in non-survivors on the day of death (*p* = 0.031). ICU, intensive care unit; PLT, platelet count

Of 180 patients, 11 (6.11 %), 5 (2.78 %), 60 (33.33 %), and 123 (68.33 %) had a prolonged PT (> 3 s), prolonged aPTT (> 10 s), decreased antithrombin concentration, and increased fibrinogen concentration, respectively, on admission to the ICU (Table 3). These coagulation abnormalities were not related to mortality. A decreased platelet count, which is reportedly relatively uncommon at the initial presentation of patients with COVID-19, was associated with non-survival in the present study. Forty-seven (26.11 %) patients with a platelet count of < 150 × 10^9^/L on admission to the ICU had higher mortality. The D-dimer concentration was higher than that shown in many previous studies [1–5, 11], and 154 (85.56 %) patients had a D-dimer concentration of > 0.5 mg/L. Even for patients without a history of cancer, diabetes, or VTE, 114 (82.61 %) had an increased D-dimer concentration. The D-dimer concentration was stratified into four levels as shown in Table 3, and a D-dimer concentration of > 0.5 mg/L on admission was significantly associated with 30 day mortality. A D-dimer concentration of > 5 mg/L was found in 62 (34.44 %) patients, and 43 (69.35 %) of them were non-survivors. The concentration of FDP, another hyperfibrinolysis-associated parameter, was elevated in 55 (61.80 %) non-survivors, which was a significantly higher proportion than survivors. To study the association between coagulation disorders and disease development, we compared parameters on the day of ICU admission versus the day of ICU discharge or death. Among 89 non-survivors, the D-dimer concentration was increased and the platelet count and antithrombin concentration were decreased significantly. In contrast, the platelet count had recovered and the fibrinogen concentration was decreased in 91 survivors on the day of ICU discharge or end of data collection compared with the day of ICU admission (Fig. 1).

**Table 3.** Coagulation abnormalities/clinical prognostic factors and mortality

Prognostic factors	Survivors		Non-survivors		*P* value
	No.	%	No.	%	
Temperature_max_ (°C)					0.291
≥39	45	25.00	51	28.33	
<39	46	25.56	38	21.11	
Duration of Low BP (h)					0.000*
0	83	46.11	25	13.89	
<72	1	0.56	25	13.89	
≥72	7	3.89	39	21.67	
Blood transfusion (ml)					0.037*
0	73	40.56	55	30.56	
≤800	13	7.22	20	11.11	
>800	6	3.33	13	7.22	
Lymphocyte					0.000*
≥1.0 × 10^9^/L	33	18.33	7	3.89	
<1.0 × 10^9^/L	58	32.22	82	45.56	
Platelet count					0.001*
≥150 × 10^9^/L	77	42.78	56	31.11	
<150 × 10^9^/L	14	7.78	33	18.33	
SIC score					0.002*
<4	85	47.22	69	38.33	
≥4	6	3.33	20	11.11	
DIC score					0.000*
<5	78	43.33	48	26.67	
≥5	13	7.22	41	22.78	
Overt DIC					0.000*
Yes	4	2.22	59	32.78	
No	87	48.33	30	16.67	
Prolongation of PT (s)					0.785
>3	6	3.33	5	2.78	
≤3	85	47.22	84	46.67	
Prolongation of aPTT (s)					0.632
>10	2	1.11	3	1.67	
≤10	89	49.44	86	47.78	
Fbg (g/L)					0.160
1.5–4	32	17.78	21	11.67	
>4	58	32.22	65	36.11	
<1.5	1	0.56	3	1.67	
D-dimer (mg/L)					0.000*
≤0.5	25	13.89	1	0.56	
>0.5 and ≤2	34	18.89	27	15.00	
>2 and <5	13	7.22	18	10.00	
>5	19	10.56	43	23.89	
FDP (mg/L)					0.000*
≤5	52	28.89	10	5.56	
>5 and ≤20	12	6.67	21	11.67	
>20	9	5.00	34	18.89	
Antithrombin (%)					0.147
≥80	20	11.11	37	20.56	
<80	29	16.11	31	17.22	
Serum calcium levels (mmol/L)					0.002*
≥1.8	70	38.89	73	40.56	
<1.8	2	1.11	16	8.89	
VTE prophylaxis					0.098
Yes	29	16.11	39	21.67	
No	62	34.44	50	27.78	

*BP* blood pressure, *SIC* sepsis-induced coagulopathy, *DIC* disseminated intravascular coagulation, *PT* prothrombin time, *aPTT* activated partial thromboplastin time, *Fbg* fibrinogen, *FDP* fibrin degradation products, *VTE* venous thromboembolism

Blood transfusion: red blood cells, platelet or fresh-frozen plasma

*Statistically significant

#### VTE

Doppler ultrasound was not performed for all patients with a hypercoagulable state. Only 26 patients underwent ultrasonography, and 19 were confirmed to have VTE with an incidence of 73.77 %. No differences were found in sex, age, or a history of cancer, diabetes, or VTE between patients with and without VTE (Table 4). Coagulation parameters and clinical features were compared in the 26 patients who underwent ultrasonography. As shown in Table 5, an abnormal platelet count, fibrinogen concentration, and D-dimer concentration on admission did not increase the incidence of VTE, and none of the adverse clinical features evaluated in this study were associated with VTE.

**Table 4.** Coagulation abnormalities and clinical prognostic factors

Characteristics	VTE		non-VTE		*P* value
	No.	%	No.	%	
Age					0.353
<60	7	26.92	4	15.38	
≥60	12	46.15	3	11.54	
Sex					0.186
Male	11	42.31	6	23.08	
Female	8	30.77	1	3.85	
Cancer history					0.444
Yes	1	3.85	1	3.85	
No	18	69.23	6	23.08	
Diabetes history					0.444
Yes	1	3.85	1	3.85	
No	18	69.23	6	23.08	
VTE history					0.536
Yes	1	3.85	0	0.00	
No	18	69.23	7	26.92	

*VTE* venous thromboembolism

**Table 5.** Coagulation abnormalities/clinical prognostic factors and VTE

Prognostic factors	VTE		non-VTE		*P* value
	No.	%	No.	%	
Temperature_max_ (°C)					0.780
≥39	12	46.15	4	15.38	
<39	7	26.92	3	11.54	
Duration of Low BP (h)					0.171
0	6	23.08	5	19.23	
<72	2	7.69	0	0.00	
≥72	11	42.31	2	7.69	
Blood transfusion (ml)					0.184
0	6	23.08	5	19.23	
≤800	8	30.77	1	3.85	
>800	5	19.23	1	3.85	
Lymphocyte					0.952
≥1.0 × 10^9^/L	3	11.54	1	3.85	
<1.0 × 10^9^/L	16	61.54	6	23.08	
Platelet count					0.143
≥150 × 10^9^/L	14	53.85	3	11.54	
<150 × 10^9^/L	5	19.23	4	15.38	
SIC score					0.258
<4	17	65.38	5	19.23	
≥4	2	7.69	2	7.69	
DIC score					0.269
<5	12	46.15	6	23.08	
≥5	7	26.92	1	3.85	
Overt DIC					0.124
Yes	9	34.62	1	3.85	
No	10	38.46	6	23.08	
Prolongation of PT (s)					
>3	0	0.00	0		
≤3	19	73.08	7	26.92	
Prolongation of aPTT (s)					0.536
>10	1	3.85	0		
≤10	18	69.23	7	26.92	
Fbg (g/L)					0.423
1.5–4	4	15.38	2	7.69	
>4	9	34.62	10	38.46	
<1.5	0	0.00	1	3.85	
D-dimer (mg/L)					0.510
≤0.5	2	7.69	0		
>0.5 and ≤2	5	19.23	5	19.23	
>2 and <5	2	7.69	3	11.54	
>5	4	15.38	5	19.23	
Antithrombin (%)					0.627
≥80	11	42.31	4	15.38	
<80	5	19.23	1	3.85	
Serum calcium levels (mmol/L)					
≥1.8	19	73.08	7	26.92	
<1.8	0	0.00	0		
VTE prophylaxis					0.78
Yes	12	46.15	4	15.38	
No	7	26.92	3	11.54	

*BP* blood pressure, *SIC* sepsis-induced coagulopathy, *DIC* disseminated intravascular coagulation, *PT* prothrombin time, *aPTT* activated partial thromboplastin time,*Fbg* fibrinogen, *VTE* venous thromboembolism

Blood transfusion: red blood cells, platelet or fresh-frozen plasma

### SIC and DIC

**Fig. 2:**
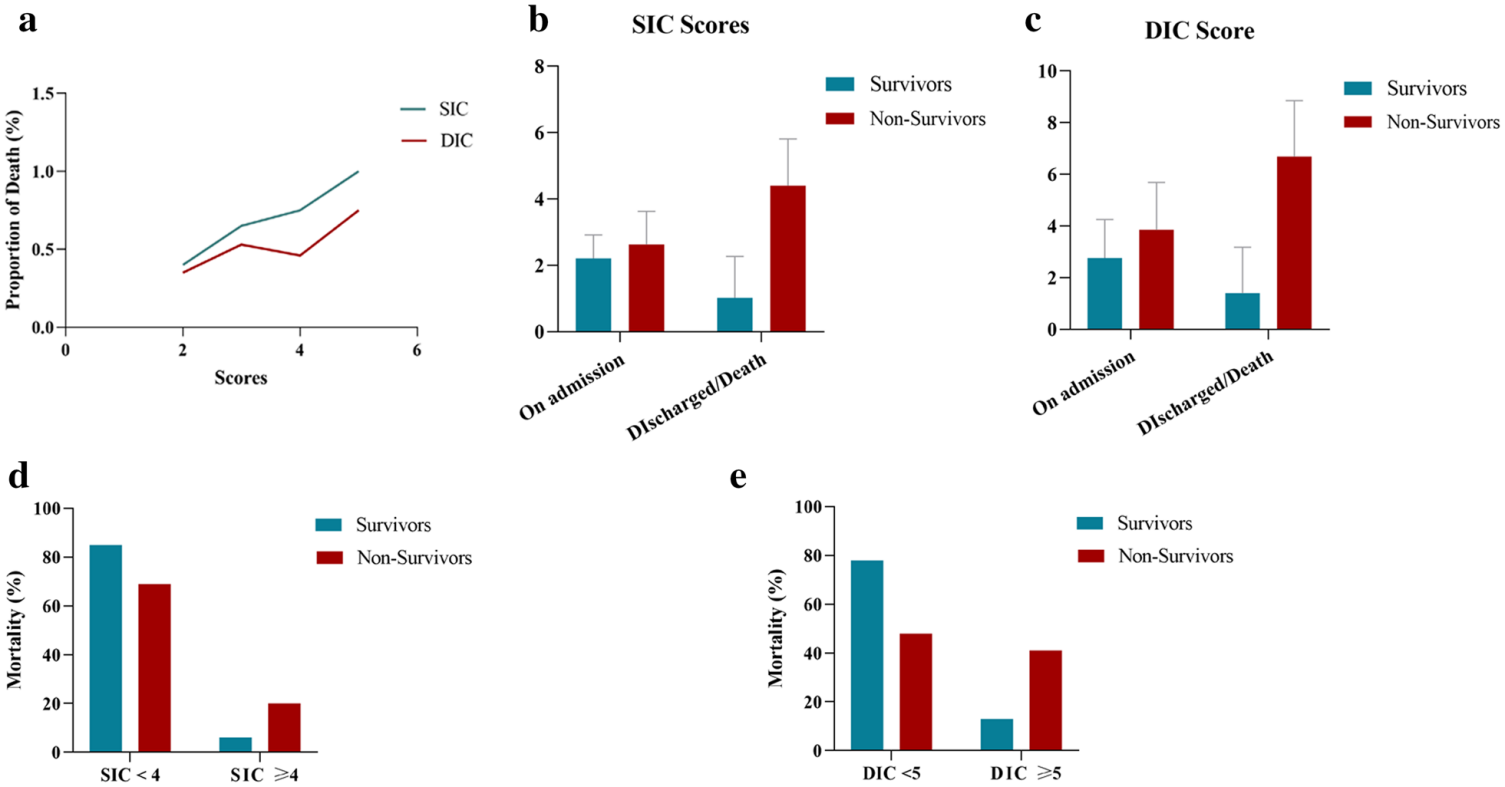
SIC and DIC scores in predicting mortality of patients with COVID-19. **a** Both the SIC and DIC scores of non-survivors were much higher than those of survivors. **b**, **c** The SIC and DIC scores were significantly higher in non-survivors on the day of death than on the day of admission to the intensive care unit (*p* = 0.000). **d** The mortality rate was significantly higher in patients with an SIC score of ≥ 4 than in those with an SIC score of < 4 (*p* = 0.002). **e** The mortality rate was significantly higher in patients with a DIC score of ≥ 5 than in those with a DIC score of < 5 (*p* = 0.000). SIC, sepsis-induced coagulopathy; DIC, disseminated intravascular coagulation

The development of SIC and DIC secondary to a profound inflammatory response may predispose to high mortality in patients with severe COVID-19. We compared the SIC and DIC scores between survivors and non-survivors (Table 3). The median SIC and DIC scores of non-survivors and survivors on admission to the ICU were 2.63 ± 1.00 and 2.21 ± 0.71 (*p* = 0.000) and 3.85 ± 1.84 and 2.76 ± 1.49 (*p* = 0.000), respectively. Both the SIC and DIC scores of non-survivors were much higher than those of survivors. The proportion of deaths increased as the SIC and DIC scores increased (Fig. 2a, b). Comparison of the SIC and DIC scores on the day of ICU admission versus the day of ICU discharge or death showed that the SIC scores significantly increased in non-survivors as the disease progressed and decreased in survivors on the day of ICU discharge (*p* < 0.05) (Fig. 2c, d). Mortality was 3.048-fold higher in patients with a SIC score of ≥ 4 than in those with a SIC score of < 4. Higher DIC scores were seen in non-survivors than survivors and also increased as the disease progressed (*p* < 0.05) (Fig. 2e, f). Mortality was 5.376-fold higher in patients with a DIC score of ≥ 5 than in those with a DIC score of < 5. Patients diagnosed with overt DIC according to the ISTH criteria had 68 % higher mortality.

### VTE prophylaxis

VTE prophylaxis was initiated in 68 (37.78 %) of 180 patients, including 12 (63.16) of 19 patients who were confirmed to have VTE during hospitalisation despite administration of enoxaparin at prophylactic doses. VTE prophylaxis in these patients was administered based on our experience treating patients with severe infection. Before patients were selected in terms of coagulation abnormalities, VTE prophylaxis was associated with neither survival nor the incidence of VTE (Tables 3 and 5). However, the association with 30-day survival was significant after patients with severe coagulopathy were selected as follows: patients with a DIC score of ≥ 5 (*p* = 0.022), D-dimer concentration of > 2 mg/L (*p* = 0.045), or confirmed overt DIC (*p* = 0.043).

### Clinical features: correlation with survival

Compared with patients in the ward, patients in the ICU have more severe clinical features and risk factors that can contribute to higher mortality or VTE incidence. We compared the body temperature, duration of low blood pressure, blood transfusion volume (total volume of red blood cells, platelet and fresh-frozen plasma), and lymphocyte count on the day of ICU admission versus the day of ICU discharge or death. The results showed that a low blood pressure duration of > 72 hours, blood transfusion of > 800 ml, and lymphocytopenia were risk factors for mortality (Table 3). Lymphocytopenia was present in 77.78 % of the patients on admission to ICU with the median lymphocyte count of 0.64 × 10^9^/L which returned to normal on the day of ICU discharge or continued to decrease until death occurred (median: 0.47 × 10^9^/L). The incidence of lymphocytopenia was 92.13 % in non-survivors, which was significantly higher than that of survivors (63.74 %).

The close relationship between serum calcium and coagulation factor IV, which is involved in haemostasis, was also studied because a serum calcium concentration was observed in more non-survivors than survivors. Of 89 non-survivors, 8.89 % of patients had a decreased serum calcium concentration while only 1.11 % had a serum calcium concentration within the normal range.

### Survival

**Fig. 3:**
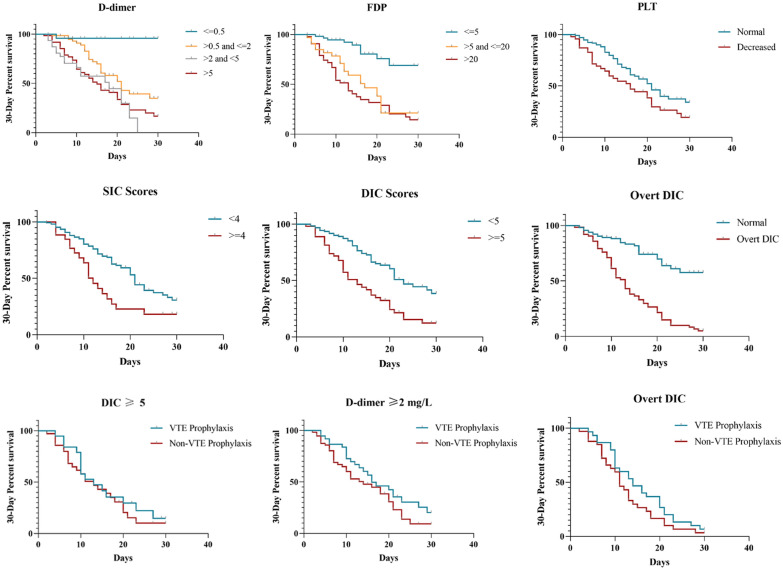
Thirty-day survival rates and associated risk factors, including increased D-dimer and FDP concentrations, platelet count of ≤ 150 × 10^9^/L, SIC score of ≥ 4, DIC score of ≥ 5 and overt DIC. The 30 day survival rate was significant after patients with severe coagulopathy were selected as follows: patients with a DIC score of ≥ 5 (*p* = 0.022), D-dimer concentration of > 2 mg/L (*p* = 0.045), or confirmed overt DIC (*p* = 0.043). FDP, fibrin degradation products; PLT, platelet count; SIC, sepsis-induced coagulopathy; DIC, disseminated intravascular coagulation; VTE, venous thromboembolism

The median follow-up period was 13 days (range, 2–60 days). The 30 day and 60 day cumulative survival rates were 51.11 % and 50.56 %, respectively, suggesting that most non-survivors died within 30 days after admission to the ICU. Factors associated with decreased 30 day survival rates included a platelet count of ≤ 150 × 10^9^/L, increased D-dimer and FDP concentrations, an SIC score of ≥ 4, a DIC score of ≥ 5, overt DIC, and VTE prophylaxis in patients with severe coagulopathy as mentioned above. Other clinical features such as an age of ≥ 60 years, low blood pressure duration of ≥ 72 hours, lymphocytopenia, and decreased serum calcium concentration could also contribute to mortality. Survival curves are shown in Fig. 3.

## Discussion

Critical illness is the main contributor to the mortality of COVID-19. This retrospective study focused on patients with severe COVID-19 in the ICU with regard to coagulation abnormalities that may contribute to higher mortality than in ward patients.

Unlike severely ill patients with sepsis, patients with COVID-19 rarely present with prolongation of the PT or aPTT, although a slightly prolonged PT has been reported in patients with severe disease [1, 2, 7, 12]. An elevated D-dimer concentration is widely accepted as a specific coagulation abnormality in patients with COVID-19. A rising D-dimer concentration suggests a hypercoagulable state and microthrombus formation, and increased rates of VTE have been reported in ICU patients with COVID-19 [11]. However, an elevated D-dimer concentration was not associated with VTE in this study. We hypothesised that a rising D-dimer concentration can result from limited microthrombosis before VTE formation. The lack of VTE screening for all patients might also explain the lack of relevance of the D-dimer concentration in the present study. The D-dimer concentrations in non-survivors continued to increase as the disease developed. A D-dimer concentration of > 0.5 mg/L on admission was significantly associated with mortality, and a higher proportion of non-survivors than survivors had a D-dimer concentration of > 5 mg/L. A dramatically increased D-dimer concentration (> 5 mg/L) may be due to overt DIC, which seems to be less common in patients with severe COVID-19 than in patients with other infections, although the mortality rate reached > 90 %. The concentration of FDP, another parameter associated with hyperfibrinolysis, was also elevated in non-survivors; this is consistent with the increased D-dimer concentration. The roles of the ISTH SIC and CDSS DIC scoring systems in predicting mortality of patients with COVID-19 were evaluated. Mortality increased as the SIC and DIC scores increased, especially when the SIC score reached ≥ 4 and the DIC score reached ≥ 5. Because both scoring systems were positively correlated with the mortality rate, further studies of larger populations are needed to determine which score can more sensitively predict mortality.

The incidence of VTE was high in patients who underwent VTE screening but was low in all 180 patients with severe disease in this study. If VTE screening had been applied, the incidence could have been even higher. VTE management in patients with COVID-19 is important, and whether VTE prophylaxis should be used remains controversial. Reports indicate that VTE prophylaxis should be considered in all patients who require hospital admission, but survival advantages have only been found in patients who meet the diagnostic criteria for SIC. Stratification by the SIC score revealed lower mortality in patients treated with heparin when the SIC score was > 4. However, anticoagulant treatment in patients with a D-dimer concentration of ≤ 1 mg/L has potential risk [5, 6, 13]. In the present study, enoxaparin for VTE prophylaxis was given to 68 (37.78 %) patients, and no difference in 30 day mortality was seen between patients with and without enoxaparin treatment. Nevertheless, stratification by the D-dimer concentration and DIC score confirmed lower mortality in patients treated with enoxaparin when the D-dimer concentration was > 2 than < 2 mg/L or when the DIC score was ≥ 5 than < 5. Our results suggested that VTE prophylaxis should be given to selected patients with a D-dimer concentration of > 2 mg/L or DIC score of ≥ 5. These results should be regarded with some degree of caution due to limitations associated with the study including lack of VTE screening for all patients by doppler ultrasound or by further tests such as von Willebrand Factor (vWF). The incidence of VTE might be higher and the association between VTE and the mortality of severe COVID-19 disease might be positive if VTE screening was performed for all patients in ICU. vWF levels is another parameter to predict VTE and might help making VTE prophylaxis earlier and more precise.

COVID-19 may predispose to not only venous but also arterial thromboembolic disease because of the effects of comprehensive factors including excessive inflammation, platelet activation, endothelial dysfunction, and stasis [7, 14]. Platelet is the initiating factor in arterial thrombosis; however, thrombocytopenia is reportedly more common in patients with severe COVID-19 [2, 3, 13, 15, 16]. Thrombocytopenia is attributed to abnormal hemostasis and DIC, and is associated with severe disease manifestation and increased mortality in patients with COVID-19 [15]. Thrombocytopenia was also proven to be a predictor of mortality in the present study. No patients in our study died of haemorrhage; instead, most patients who underwent ultrasonography were confirmed to have VTE. Therefore, platelet activation might occur in association with COVID-19.

Moreover, according to our clinical observations, more non-survivors than survivors presented with a low serum calcium concentration. We speculated that most critically ill patients received a massive blood transfusion, which promoted the chelation of ionised calcium by citric acid and resulted in hypocalcaemia. We then studied the correlation between the blood transfusion volume and serum calcium concentration, and a statistically significant correlation was found (data not shown). Therefore, hypocalcaemia might be a predictor of severe disease. Whether hypocalcaemia impacts the cascade pathways of coagulation requires further study. Other clinical features found to be indicators of mortality were lymphocytopenia, a low blood pressure duration of > 72 hours, and a blood transfusion volume of > 800 ml during hospitalisation.

In conclusion, a D-dimer concentration of > 0.5 mg/L on admission is a risk factor for severe disease. An ISTH SIC score of > 4 and CDSS DIC score of > 5 can be used to predict mortality. Thromboembolic prophylaxis can reduce mortality only in patients with a D-dimer concentration of > 2 mg/L or DIC score of ≥ 5. Further prospective studies are needed to determine whether antiplatelet therapy can work with anticoagulant therapy to improve clinical outcomes.

## Data Availability

Not applicable.

## Abbreviations

COVID-19: Coronavirus disease 2019
SIC: Sepsis-induced coagulopathy
DIC: Disseminated intravascular coagulation
ICU: Intensive care unit
VTE: Venous thromboembolism
ISTH: International Society on Thrombosis and Haemostasis
PT,: Prothrombin time
aPTT: Activated partial thromboplastin time
FDP: Fibrin degradation product
CDSS: Chinese DIC scoring system
